# Синдром Кальмана у монозиготных близнецов как изолированное проявление дефекта гена SOX10

**DOI:** 10.14341/probl12789

**Published:** 2021-09-07

**Authors:** Е. Б. Фролова, В. М. Петров, Е. В. Васильев, Н. A. Макрецкая, О. В. Пилипенко, А. Н. Тюльпаков

**Affiliations:** Национальный медицинский исследовательский центр здоровья детей; Национальный медицинский исследовательский центр эндокринологии; Национальный медицинский исследовательский центр эндокринологии; Медико-генетический научный центр им. Н.П. Бочкова; Детская городская клиническая больница №1; Медико-генетический научный центр им. Н.П. Бочкова; Республиканская детская клиническая больница

**Keywords:** нейросенсорная тугоухость, синдром Кальмана, SOX10, клинический случай

## Abstract

На сегодняшний день известно более 30 генов, дефекты в которых могут приводить к развитию врожденного изолированного гипогонадотропного гипогонадизма (иГГ), роль еще более 10 генов изучается, и, тем не менее, до 50% случаев иГГ все еще не находят молекулярно-генетического объяснения.

В структуре иГГ выделяют ряд синдромальных форм — сочетаний гипогонадизма с нерепродуктивными проявлениями. Так, для дефектов некоторых генов, кодирующих факторы нейрональной миграции, известно сочетание синдрома Кальмана с нейросенсорной тугоухостью, среди пациентов с данным фенотипом чаще других выявляются дефекты в CHD7, SOX10. Однако для дефектов генов факторов нейрональной миграции характерна широкая вариабельность фенотипических проявлений, что связывают с влиянием эпигенетических механизмов. У носителей мутации в пределах одной семьи могут отсутствовать как нерепродуктивные проявления, так и гипогонадизм. В данной статье представлено описание случая синдрома Кальмана у монозиготных близнецов, обусловленного ранее не описанной гетерозиготной мутацией c.462C>G p.I154M в гене SOX10, не ассоциированного с нейросенсорной тугоухостью, унаследованной от отца, имеющего в фенотипе лишь аносмию и не страдающего гипогонадизмом. Мутация выявлена в ходе полноэкзомного секвенирования. Это первое подобное наблюдение в России, указывающее, с одной стороны, на необходимость обследования пациентов с синдромом Кальмана на дефекты SOX10 в числе прочих факторов нейрональной миграции и дифференцировки, с другой стороны — на перспективность проведения полноэкзомного секвенирования в группе пациентов с недифференцированным иГГ.

## Abstract (en)

More than 30 genes are known to take part in hypothalamic-pituitary-gonadal axis development at the date and role of more than 10 other genes is studied. Despite it about 50% of isolated hypogonadotropic hypogonadism cases still have no molecular genetic explanation.

A number of specific associations between iHH and different not-reproductive manifestations called syndromic forms are distinguished in general group of iHH. For example, the combination of Kalmann syndrome with sensorineural hearing loss is known as manifestation for defects of some genes encoding factors of neuronal migration; in patients with this phenotype CHD7, SOX10 genes defects are most frequent. However, defects in the genes of neuronal migration factors are characterized by a wide variability of phenotype, which is explained by the epigenetic mechanisms influence. Carriers of the mutation within the same family may lack some non-reproductive manifestations as well as hypogonadism.

Here we present a case of Kalmann syndrome in monozygous twins, caused by a previously not described heterozygous mutation c.462C> G: p.I154M in the SOX10 gene in the absence of sensorineural hearing loss. The mutation was inherited from a father who has only isolated anosmia in the phenotype. This mutation was identified during full exome sequencing. This unique observation for Russia shows on the one hand expediency to check SOX10 sequence in addition to the other factors of neuronal migration and differentiation and, on the other hand, the prospect of full exome sequencing in a group of patients with undifferentiated iHH.

## ВВЕДЕНИЕ

Изолированный гипогонадотропный гипогонадизм (иГГ) — генетически гетерогенная группа заболеваний, объединенных общей клинической картиной: отсутствие признаков полового развития или незавершенный пубертат к возрасту 18 лет в сочетании с низкими уровнями гонадотропинов и половых стероидов в крови и нормальной продукцией остальных гормонов передней доли гипофиза.

Механизмы развития иГГ можно условно разделить на три группы: нарушение миграции в гипоталамус нейронов, продуцирующих гонадотропин-рилизинг-гормон (ГнРГ), нарушение синтеза или активности ГнРГ и, наконец, резистентность к ГнРГ гонадотрофов передней доли гипофиза. Фенотипически все пациенты с иГГ подразделяются на две группы: с нарушениями обоняния (синдром Кальмана (СК)) и нормосмическим ГГ. ­Фенотип СК ассоциирован с дефектами генов нейрональной миграции [1].

На сегодняшний день известно более 30 генов, дефекты в которых приводят к развитию заболевания, и, тем не менее, до 50% случаев иГГ все еще не находят молекулярно-генетического объяснения [1]. Внедрение в повседневную клиническую практику секвенирования следующего поколения (NGS) положило начало новому этапу в изучении молекулярно-генетической природы иГГ, значительно упростив и ускорив обследование. В группе пациентов с клинической картиной иГГ, не имеющих дефектов генов-кандидатов по NGS, целесообразно продолжение клинического и молекулярно-генетического обследования, в частности — полноэкзомного секвенирования.

В структуре заболевания выделяют ряд синдромальных форм — сочетаний иГГ с нерепродуктивными проявлениями: неврологическими нарушениями (бимануальные гиперкинезы при нарушении развития проводящих путей, врожденные лейкодистрофии), нейросенсорной тугоухостью, аномалиями опорно-двигательного аппарата, агенезией почки и множественными пороками развития (сочетание нескольких пороков развития, обусловленных дефектом одного гена, как и при полной CHARGE-ассоциации, когда все пороки обусловлены дефектом гена CHD7). Наличие в фенотипе таких нерепродуктивных патологий могло бы стать ключом в поиске генов-кандидатов. Так, у 5% пациентов с СК отмечается снижение слуха, а для сочетания нейросенсорной тугоухости с иГГ известно, что в 30% случаев в его основе лежат мутации в гене SOX10 и в 30–40% — в гене CHD7 [2]. У большинства ранее описанных пациентов с дефектами SOX10 отмечено снижение слуха [2–7]. Однако для иГГ характерно отсутствие четкой корреляции генотип/фенотип, велик вклад в развитие фенотипа эпигенетических механизмов, и наличие характерных нерепродуктивных проявлений непостоянно [1]. В данной статье мы описываем случай СК у монозиготных близнецов, обусловленного гетерозиготной мутацией c.462C>G:p.I154M в гене SOX10, не ассоциированного с нарушениями слуха. Мутация выявлена в ходе полноэкзомного секвенирования.

## ОПИСАНИЕ КЛИНИЧЕСКОГО СЛУЧАЯ

Семейный анамнез по эндокринным заболеваниям не отягощен. Со слов матери, у отца отмечается аносмия.

Пробанды от третьей беременности, данная беременность двойней, протекала без особенностей, роды на 36-й неделе. При рождении масса 2600 г и 2630 г, длина 46 см и 47 см. При рождении у обоих мальчиков яички в мошонке отсутствовали. В раннем возрасте осмотрены урологом, констатирован высокий кремастерный рефлекс с двух сторон.

У обоих пробандов отмечается аносмия.

Впервые обратились к эндокринологу в возрасте 14 лет с жалобами на неудовлетворительные темпы полового развития, по проведенному обследованию: ­пробанд 1: рост 160 см, вес 50 кг, половое развитие Таннер II (G1P2); яички в мошонке по 1 мл, костный возраст 14 лет, лютеинизирующий гормон (ЛГ) 0,1 Ед/л, фолликулостимулирующий гормон (ФСГ) 0,4 Ед/л, тестостерон 0,7 нмоль/л, антимюллеров гормон (АМГ) 60,64 нг/мл (3,8–159,8), ингибин В 45,9 пг/мл (35–475).

Пробанд 2: рост 161 см, вес 50 кг, костный возраст 14 лет, объем гонад 1 мл, ЛГ 0,1 ед/л, ФСГ 0,43 ед/л, тестостерон 0,8 нмоль/л, АМГ 52,12 нг/мл (3,8–159,8), ингибин В 44,5 пг/мл (35–475).

Проведена проба с люлиберином, у обоих пробандов в ходе пробы получены допубертатные значения гонадотропинов. Обоим пробандам проведен курс терапии хорионическим гонадотропином человека (ХГЧ) по схеме: ХГЧ 1,5 тыс. Ед 2 раза в неделю N 20. Через 1 мес повторно проведен курс ХГЧ 2 тыс. Ед 2 раза в неделю N10, по завершении второго курса тестостерон пробанда 1 — 0,721 нмоль/л, пробанда 2 — 1,24 нмоль/л, объем гонад 4 мл, что не противоречит диагнозу ГГ. В связи с неудовлетворительным ответом на терапию ХГЧ у обоих братьев начата терапия препаратами тестостерона с наращиванием дозы со 100 мг на введение до 250 мг на введение каждые 28 дней в течение 6 мес, далее 1 год в дозе 250 мг/28 дней, получен ответ в виде прогрессии полового развития до 4-й стадии по Таннеру в течение 12 мес, отмечено увеличение объема тестикул до 8 мл, закрытие зон роста по данным рентгенограммы кисти. Конечный рост пробанда 1 — 172 см, пробанда 2 — 174 см. В дальнейшем семья отказалась от терапии препаратами тестостерона, спустя 12 мес от последней инъекции вновь отмечены допубертатные значения тестостерона и гонадотропинов у обоих пробандов (уровни тестостерона 0,59 и 0,73 нмоль/л у пробанда 1 и 2 соответственно), объем тестикул 4 мл.

Молекулярно-генетическое исследование проведено методом высокопроизводительного параллельного секвенирования. Использовалась разработанная в отделении наследственных эндокринопатий ФГБУ «НМИЦ эндокринологии» панель праймеров Ion Ampliseq Custom DNA Panel (Life technologies, США), охватывающая кодирующие области следующих генов: CHD7, DNMT3L, DUSP6, FGF17, FGF8, FGFR1, FLRT3, GNRH1, GNRHR, HS6ST1, IL17RD, INSL3, KAL1, KISS1, KISS1R, LHB, NELF, POLR3B, PROKR2, RBM28, SEMA3A, SPRY4, TACR3, WDR11, GREAT, TAC3, KAL4, NR0B1, POLR3A, MKRN3. Секвенирование осуществлялось на полупроводниковом секвенаторе PGM (Ion Torrent, Life Technologies, США).

Значимых изменений последовательности 30 включенных генов не выявлено. Проведено полноэкзомное секвенирование, выявившее гетерозиготную мутацию в гене SOX10, мутация ранее не описана, с неопределенной патогенностью. Эта же мутация выявлена у отца пробандов.

Нарушений пигментации у пробандов нет, снижения слуха по результатам аудиометрии не выявлено.

## ОБСУЖДЕНИЕ

Ген SOX10 кодирует фактор транскрипции, участвующий в развитии нервного гребня и олигодендроцитов, периферических нервов, дифференцировке меланоцитов. Относится к семейству SOX-генов, объединенных идентичным с SRY-геном ДНК-связывающим доменом, известным как HMG (high mobility group).

В 1998 г. V. Pingault и соавт. выделили ДНК гена SOX10, кодирующую 466 аминокислот с высококонсервативным HMG-доменом, и установили, что ген SOX10 состоит из 5 экзонов [8]. Используя нозерн-блоттинг, было показано, что мРНК SOX10 выявляется в головном мозге плода, сердце, мозге, тонкой и толстой кишке взрослого. У мышей экспрессия SOX10 показана в периферической нервной системе, а наиболее выражена в тройничном, коленчатом и акустическом ядрах ствола головного мозга [9–12].

К. Kuhlbrodt и соавт. (1998) клонировали SOX10 крысы и показали, что в ходе развития эмбриона его экспрессия, впервые выявляясь в формирующемся нервном гребне, обеспечивает формирование периферической нервной системы и в конечном итоге — дифференцировку клеток в шванновские клетки. В центральной нервной системе SOX10 экспрессируется в предшественниках глиальных клеток и позднее выявляется в олигодендроцитах мозга взрослого. Белок SOX10 реализует свое действие путем активации фактора транскрипции MITF (melanocyte inducing transcription factor — фактор транскрипции, индуцирующий развитие меланоцитов) в синергизме с фактором транскрипции PAX3 (paired box3).

Известна ассоциация мутаций в гене SOX10 с развитием различных форм синдрома Варденбурга–Шаха (ВШ — сочетание нарушений пигментации с нейросенсорной тугоухостью) [1][8][9][12]. Выделяют тип 4С (ВШ4С), сочетающийся с болезнью Гиршпрунга, а также особый тип, ассоциированный с тяжелой периферической полинейропатией, лейкодистрофией и болезнью Гиршпрунга. Роль SOX10 в дифференцировке нейронов, продуцирующих ГнРГ, описана на животных моделях K. Whitlock [13].

Впервые предположение о возможной связи мутаций в SOX10 с СК возникло в ходе исследования МР-изображений головы, полученных в группе пациентов с фенотипом синдрома ВШ, исследование проводилось в связи с тугоухостью с целью оценки морфологии височных костей. У 16 из 17 обследованных была отмечена аплазия или гипоплазия обонятельных луковиц. У одного из пациентов был ранее установлен диагноз иГГ [3].

V. Pingault и соавт. (2013) исследовали ген SOX10в группе из 17 пациентов с ГГ и аносмией (СК) и как минимум одним симптомом, характерным для синдрома ВШ (нарушение пигментации и/или нейросенсорная тугоухость). В ходе исследования у 6 пациентов были выявлены гетерозиготные мутации в гене SOX10(30% обследованных). Далее ген SOX10 исследован в группе из 86 пациентов с СК, у 20 из которых отмечались различные ассоциированные нерепродуктивные аномалии. У 2 пациентов были выявлены гетерозиготные мутации в SOX10, у одного из пациентов был снижен слух, у второго слух был в норме [3]. Автор отметила, что вероятна гиподиагностика иГГ и аносмии в группе больных с синдромом ВШ, который, как правило, диагностируется в раннем возрасте, и пациенты активно не жалуются на аносмию и задержку полового развития [6][11].

V. Pingault и соавт. (2013) выявили экспрессию SOX10 в предшественниках обонятельных нервов в ходе их развития у мышей и человека. Авторами была показана практически полная аплазия предшественников обонятельных луковиц у SOX10-нокаутных мышей и неправильное развитие нейронов, продуцирующих ГнРГ, что подтверждало связь дефектов SOX10и СК [3][14].

За прошедшие с первого описания ассоциации СК с дефектами SOX10 7 лет выявлено более 20 мутаций в гене SOX10, приводящих к развитию гипогонадизма. У большинства описанных пациентов отмечается нарушение слуха (уни- или билатеральное снижение слуха, чаще — на высоких частотах) [2–7].

Интересен тот факт, что среди пациентов со всеми типами синдрома ВШ гипогонадизм описан всего в нескольких случаях. Вероятно, это связано с гиподиагностикой этого состояния, отчасти объяснимой возрастом пациентов на момент постановки диагноза. Однако в ходе исследований на мышиных моделях установлено, что при мутациях, характерных для синдрома ВШ, в гетерозиготном положении у особей, сформировавших фенотип синдрома ВШ (аганглиоз кишки и нарушения пигментации), не отмечалось проявлений СК. И только у гомозигот по мутантному аллелю отмечена гипоплазия обонятельных луковиц [11].

В 2016 г. впервые описан случай спонтанного стойкого (по крайней мере в течение 10 лет) восстановления синтеза гонадотропинов у мужчины с мутацией в SOX10 [6].

W. Dai и соавт., проведя полноэкзомное секвенирование в смешанной группе из 145 пациентов с ГГ, 60% из которых имели фенотип СК, обнаружили 3 новых мутации в экзоне 2 гена SOX10, у одного из пробандов — в гомозиготном положении, и провели функциональное исследование выявленных мутаций. Степень нарушений слуха варьировала от полной глухоты (у носителя гомозиготной мутации) до нормального слуха (у носителя гетерозиготной мутации с остаточной активностью фермента 30%). У третьего пробанда с гетерозиготной мутацией с полной утратой функции продукта мутантного аллеля отмечалось унилатеральное снижение слуха. Авторами выдвинуто предположение о связи остаточной активности фермента с наличием тугоухости [7].

У наших пациентов выявлена гетерозиготная миссенс-мутация c.462C>G:p.I154M, унаследованная от отца, который страдает аносмией. В литературе есть описания случаев гипогонадизма, при которых у родителей отмечаются лишь нерепродуктивные проявления дефекта SOX10 [3]. В качестве объяснения такой вариабельности фенотипа при одной и той же мутации предложен вклад эпигенетических механизмов [3].

## ЗАКЛЮЧЕНИЕ

Таким образом, нами представлен клинический случай ранее не описанной гетерозиготной мутации c.462C>G:p.I154M в гене SOX10 у монозиготных сибсов с СК. Данное наблюдение демонстрирует вариабельность фенотипа при синдромальных формах ГГ, которые могут проявляться лишь отдельными клиническими компонентами (в нашем случае: гипогонадизм и аносмия без других проявлений синдрома ВШ), и подчеркивает роль полноэкзомного секвенирования в диагностике редких вариантов ГГ.

## ДОПОЛНИТЕЛЬНАЯ ИНФОРМАЦИЯ

Информация о финансировании. Работа выполнена при содействии Фонда поддержки и развития филантропии «КАФ».

Конфликт интересов Авторы декларируют отсутствие явных и потенциальных конфликтов интересов, связанных с публикацией настоящей статьи.

Согласие пациента. Добровольные информированные согласия пациентов и их законных представителей на публикацию в журнале «Проблемы эндокринологии» получены.

Участие авторов: Фролова Е.Б. — анализ полученных данных, написание текста статьи; Пилипенко О.В. — сбор материала; Макрецкая Н.А. — анализ полученных данных; Васильев Е.В. — проведение молекулярно-генетического исследования; Петров В.М. — проведение молекулярно-генетического исследования; Тюльпаков А.Н. — концепция и дизайн исследования, анализ полученных данных, проведение молекулярно-генетического исследования.

Благодарности. Выражаем благодарность Фонду поддержки и развития филантропии «КАФ» за помощь в проведении исследования.
